# C-CARE: comparing two years of anaphylaxis in children treated at the Montreal Children’s Hospital

**DOI:** 10.1186/1710-1492-10-S1-A6

**Published:** 2014-03-03

**Authors:** Elana Hochstadter, Ann Clarke, Sebastien LaVieille, Reza Alizadehfar, Christopher Mill, Yuka Asai, Harley Eisman, Moshe Ben-Shoshan

**Affiliations:** 1 Department of Pediatrics, London Children’s Hospital, Western University, London, ON, Canada; 2 Division of Allergy and Clinical Immunology, Department of Medicine, McGill University Health Center, Montreal, Quebec, Canada; 3 Division of Clinical Epidemiology, Department of Medicine, McGill University Health Center, Canada; 4 Food Directorate, Health Canada, Ottawa, ON, Canada; 5 Division of Pediatric Allergy and Immunology, Department of Pediatrics, Montreal Children’s Hospital, McGill University Health Center, Canada; 6 Division of Dermatology, Department of Medicine, McGill University Health Centre, Canada

## Background

Studies suggest that rates of anaphylaxis, the most severe form of an allergic reaction, are increasing in Pediatric Emergency Departments (PED). Significant gaps still exist regarding the prevalence, triggers and temporal trends of anaphylaxis in Canada. The Cross-Canada Anaphylaxis Registry (C-CARE) was created with the primary goal of determining the societal burden of anaphylaxis in Canada; our aim was to use C-CARE to examine temporal trends in anaphylaxis rates.

## Methods

Over a 2-year period (April 2011 to April 2013), children presenting to the Montreal Children’s Hospital PED with anaphylaxis were recruited. The treating physician documented characteristics and triggers of anaphylactic reactions using a standardized data entry form. Charts of all PED patients were reviewed to identify anaphylactic cases that were missed in prospective recruitment.

## Results

Among 81,677 PED visits in Year 1, 168 anaphylaxis cases were identified (0.21%) versus 218 anaphylaxis cases among 78,650 PED visits in Year 2 (0.27%), yielding a difference of 0.06% (95% CI, 0.02%, 0.12%) between the two years. The median age of anaphylaxis was 4.8 years (IQR: 2.3-10.1) in Year 1 and 5.9 years (IQR 2.1-11.1) in Year 2. There was a slightly higher male predominance in cases of anaphylaxis in both Year 1 (51.8%; 95% CI, 44%, 59.5%) and Year 2 (61%; 95% CI, 54.2%, 67.4%). The major triggers in both years were food allergens (87.5% and 80.6% respectively) with peanut being the most predominant (29.5% and 20.6%) followed by tree nuts (15.5% and 14.8%). Severe anaphylaxis (hypoxia, cyanosis, circulatory collapse, incontinence or neurological symptoms) was found in 7.1% (95% CI, 3.9%, 12.4%) of patients from Year 1 versus 3.7% (95% CI, 1.7%, 7.4%) in Year 2.

## Conclusions

There was a greater rate of anaphylaxis in the second year of the C-CARE study. In both years, food allergens were found to be the most common trigger with peanuts being the most common food. These results are comparable to the increasing rates of anaphylaxis seen in other westernized countries. Future studies in the Montreal Children’s Hospital as well as in other centers are required to establish temporal trends in anaphylaxis rates, triggers and reaction characteristics.

